# Chloroplast-to-apoplast relocalization of MOC1 strengthens plant vascular immunity

**DOI:** 10.1093/hr/uhag046

**Published:** 2026-02-19

**Authors:** Xinya Du, Yijie Liu, Sai Yuan, Pengyue Li, Yingshuang Liu, Yang Lin, Meng Yuan, Jiatao Xie, Jiangsen Cheng, Yanping Fu, Daohong Jiang, Xiao Yu, Bo Li

**Affiliations:** State Key Laboratory of Agricultural Microbiology, Huazhong Agricultural University, Wuhan, Hubei 430070, China; The Provincial Key Lab of Plant Pathology of Hubei Province, College of Plant Science and Technology, Huazhong Agricultural University, Wuhan, Hubei 430070, China; Hubei Hongshan Laboratory, Wuhan, Hubei 430070, China; State Key Laboratory of Agricultural Microbiology, Huazhong Agricultural University, Wuhan, Hubei 430070, China; The Provincial Key Lab of Plant Pathology of Hubei Province, College of Plant Science and Technology, Huazhong Agricultural University, Wuhan, Hubei 430070, China; Hubei Hongshan Laboratory, Wuhan, Hubei 430070, China; State Key Laboratory of Agricultural Microbiology, Huazhong Agricultural University, Wuhan, Hubei 430070, China; The Provincial Key Lab of Plant Pathology of Hubei Province, College of Plant Science and Technology, Huazhong Agricultural University, Wuhan, Hubei 430070, China; Hubei Hongshan Laboratory, Wuhan, Hubei 430070, China; State Key Laboratory of Agricultural Microbiology, Huazhong Agricultural University, Wuhan, Hubei 430070, China; The Provincial Key Lab of Plant Pathology of Hubei Province, College of Plant Science and Technology, Huazhong Agricultural University, Wuhan, Hubei 430070, China; Hubei Hongshan Laboratory, Wuhan, Hubei 430070, China; Hubei Hongshan Laboratory, Wuhan, Hubei 430070, China; National Key Laboratory of Crop Genetic Improvement, Huazhong Agricultural University, Wuhan 430070, China; The Provincial Key Lab of Plant Pathology of Hubei Province, College of Plant Science and Technology, Huazhong Agricultural University, Wuhan, Hubei 430070, China; Hubei Hongshan Laboratory, Wuhan, Hubei 430070, China; National Key Laboratory of Crop Genetic Improvement, Huazhong Agricultural University, Wuhan 430070, China; State Key Laboratory of Agricultural Microbiology, Huazhong Agricultural University, Wuhan, Hubei 430070, China; The Provincial Key Lab of Plant Pathology of Hubei Province, College of Plant Science and Technology, Huazhong Agricultural University, Wuhan, Hubei 430070, China; Hubei Hongshan Laboratory, Wuhan, Hubei 430070, China; State Key Laboratory of Agricultural Microbiology, Huazhong Agricultural University, Wuhan, Hubei 430070, China; The Provincial Key Lab of Plant Pathology of Hubei Province, College of Plant Science and Technology, Huazhong Agricultural University, Wuhan, Hubei 430070, China; The Provincial Key Lab of Plant Pathology of Hubei Province, College of Plant Science and Technology, Huazhong Agricultural University, Wuhan, Hubei 430070, China; State Key Laboratory of Agricultural Microbiology, Huazhong Agricultural University, Wuhan, Hubei 430070, China; The Provincial Key Lab of Plant Pathology of Hubei Province, College of Plant Science and Technology, Huazhong Agricultural University, Wuhan, Hubei 430070, China; Hubei Hongshan Laboratory, Wuhan, Hubei 430070, China; State Key Laboratory of Agricultural Microbiology, Huazhong Agricultural University, Wuhan, Hubei 430070, China; The Provincial Key Lab of Plant Pathology of Hubei Province, College of Plant Science and Technology, Huazhong Agricultural University, Wuhan, Hubei 430070, China; Hubei Hongshan Laboratory, Wuhan, Hubei 430070, China; State Key Laboratory of Agricultural Microbiology, Huazhong Agricultural University, Wuhan, Hubei 430070, China; The Provincial Key Lab of Plant Pathology of Hubei Province, College of Plant Science and Technology, Huazhong Agricultural University, Wuhan, Hubei 430070, China; Hubei Hongshan Laboratory, Wuhan, Hubei 430070, China

## Abstract

Pathogenic bacteria deploy biofilm as a key virulence factor to cause plant vascular diseases, which are devastating to global agricultural practices. Extracellular DNA (eDNA) constitutes the backbone of bacterial biofilm and is key to biofilm stability, thereby representing as an attractive therapeutic target. Here, we engineered the plant chloroplast-localized Holliday junction (HJ) resolvase MOC1 by replacing its native chloroplast transit peptide with a secretory signal, successfully relocating it to the apoplast. Transgenic tomato and rice expressing secreted MOC1 exhibited robust resistance to bacterial wilt and bacterial blight, respectively, without growth or yield penalties. Additionally, we implemented bacterial pathogen-inducible promoters to achieve precisely spatial and temporal control over the resistance trait. Secreted MOC1 degrades eDNA *in situ*, disrupts biofilm architecture, and markedly reduces bacterial colonization and systemic spread. Our work presents a novel strategy for controlling vascular diseases by engineering plant HJ resolvases to disrupt biofilms. This approach provides a new blueprint for molecular resistance breeding and disease resistance gene exploration.

## Introduction

Plant vascular pathogenic bacteria, such as *Ralstonia solanacearum* and *Xanthomonas oryzae pv.oryzae*, cause devastating losses in many economically important crops [1–3]. Effective control of these pathogens remains challenging due to their ability to persist in soil for extended periods, their broad host range, and the rapid systemic wilting they induced by colonizing the xylem vessels and disrupting water transport [3]. Therefore, developing integrative and sustainable management strategies to combat vascular diseases is essential for ensuring the crop production, which remains as a critical research frontier.

Improvement of plant disease resistance through genetic engineering offers a highly efficient and cost-effective long-term strategy. Significant progress has been made in developing germplasm resistant to *Ralstonia* and *Xanthomonas* through the application of engineered immune genes and precision genome-editing technologies [4–7]. The executor-type resistance gene Bs4C from pepper driven by the *R. solanacearum*-induced promoter was introduced into tobacco, which exhibited improved resistance to bacterial wilt [8]. Heterologous expression of flagellin-sensitive 2 (FLS2) from soybean and elongation factor-Tu receptor (EFR) from *Arabidopsis* in tomato conferred immunity against *R. solanacearum* [6, 9–11]. Furthermore, gene editing of the promoter region of *SWEET* genes in rice enabled the development of germplasm resistant to both bacterial blight and bacterial leaf streak [12].

A profound understanding of pathogen biology is essential for developing targeted disease control strategies. Biofilm is a central virulence strategy in the infection cycle of both animal and plant pathogenic bacteria [13]. These structured communities provide robust protection against environmental stresses, host defenses, and antimicrobial agents [3, 14]. During infection, *R. solanacearum* and *X. oryzae* colonize xylem vessels, where biofilm formation represents not only merely a survival mechanism but also a central pathogenic lifestyle [15]. Targeting biofilm has been shown to be an effective strategy for controlling bacterial disease in mammals and plants [16, 17]. The structural integrity of biofilm depends on extracellular DNA (eDNA) lattices that contain Holliday junction (HJ)-like structure networks [18–21]. In the previous study, we have demonstrated that HJ resolvase RuvC from *R. solanacearum* is able to cleave HJ-like structures to disrupt the biofilm eDNA lattice, thereby facilitating biofilm dispersal. It has been further shown that heterologous expression of secreted RuvC significantly enhances resistance to vascular disease in both tomato and rice.

Plant MOC1 is a ubiquitously present HJ resolvase that localizes to chloroplasts and possesses a catalytic domain homologous to bacterial RuvC. This enzyme is essential for chloroplast nucleoid segregation in *Arabidopsis thaliana* by eliminating HJ structures [22–24]. Previously, we demonstrated that purified recombinant tomato and rice MOC1 expressed in *Escherichia coli* strongly inhibits bacterial biofilm formation by *in vitro* assay [18]. However, MOC1 possesses an N-terminal chloroplast transit peptide (CTP) instead of a secretion signal and is imported into chloroplast, whereas bacterial biofilms assemble at the apoplast, where the MOC1 protein could not reach.

In this work, to exploit the HJ enzymatic activity of MOC1 against the biofilm of vascular pathogens, we engineered tomato and rice MOC1 that are secreted into the apoplast. Transgenic tomato and rice-expressing engineered MOC1 exhibit robust resistance to bacterial wilt or bacterial blight, respectively. To achieve more targeted biofilm destruction and more accurate disease suppression, we replaced the original constitutive promoter with a pathogen-inducible promoter. This approach thereby balances improved disease resistance with minimal fitness costs. Collectively, our results confirm the feasibility of biofilm-targeted strategies for controlling bacterial diseases.

## Results

### Design and apoplast localization of MOC1 proteins

**Figure 1:**
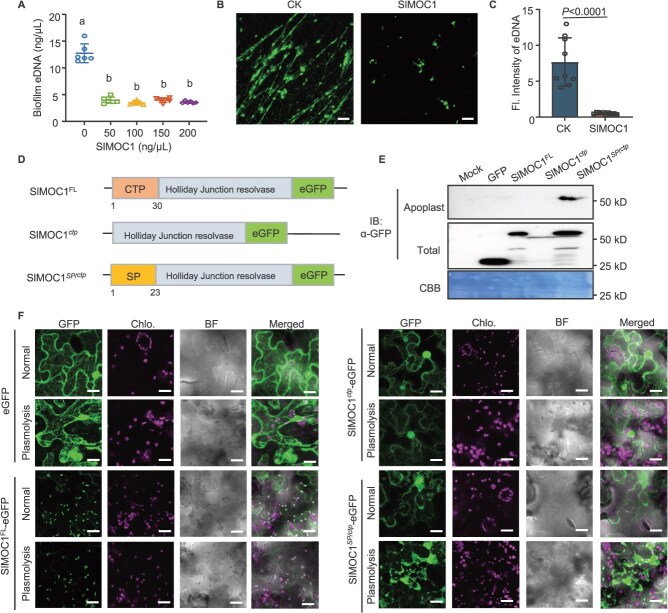
Localization of WT and engineered tomato MOC1. (A) Recombinant His-SlMOC1 efficiently degrades eDNA released by *R. solanacearum*. Data are presented as mean values ± SD (*n* = 6 biological replicates). Different letters indicate significant differences (one-way ANOVA, Tukey’s test, *P* < 0.05). (B–C) Disruption of the eDNA network in *R. solanacearum* biofilm by His-SlMOC1. The biofilm was treated with 200 ng/μl His-SlMOC1 and eDNA stained with PicoGreen (B). Scale bar, 10 μm. Fluorescence intensity of the stained eDNA (C) was quantified by ImageJ. Data are presented as mean values ± SD (*n* = 9 images). The *P-*value indicates significant differences determined by one-tailed Student’s *t*-test. (D) Scheme of various SlMOC1 constructs fused with eGFP. The N-terminal numerical labeling represents the position of different localization peptides. CTP represents chloroplast transit peptide; SP represents secretory signal peptide. (E) Western blot analysis showing the SlMOC1*^SP/ctp^-*eGFP protein is present in the apoplast of *N. benthamiana*. Total or apoplastic proteins were extracted and probed with α-GFP, and the loading was shown by Coomassie brilliant blue (CBB) staining. (F) Subcellular localization of SlMOC1^FL^, SlMOC1*^ctp^*, and SlMOC1*^SP/ctp^* fused with eGFP in tobacco leaves with or without plasmolysis. The vector that only contains eGFP was used as control. BF indicates the bright field. Scale bars, 20 μm. The above experiments were repeated three times with similar results.

Plant MOC1 comprises two primary domains: an N-terminal CTP and a C-terminal HJ resolvase domain [18, 22]. To determine whether SlMOC1 can disrupt the eDNA released *R. solanacearum*, we purified the recombinant His-SlMOC1 protein and demonstrated that it prominently reduced the eDNA content in the bacterial liquid culture (Fig. 1A). Correspondingly, the lattice structure of eDNA was significantly destroyed after His-SlMOC1 treatment (Fig. 1B). This phenomenon was further validated by quantifying the fluorescence intensity of eDNA (Fig. 1C). These results indicate that SlMOC1 can inhibit *R. solanacearum* biofilm formation by degrading the structure of eDNA. In order to engineer MOC1 for apoplastic secretion the native N-terminal CTP of SlMOC1 was replaced with the secretory signal peptide (SP) of tomato pathogenesis-related protein 1 (SlPR1) to generate SlMOC*^SP/ctp^* (Fig. 1D; Supplementary Table S1). When transiently expressed in *Nicotiana benthamiana*, the SlMOC1*^SP/ctp^-*enhanced green fluorescent protein (eGFP) fusion protein was significantly enriched in the apoplastic fluids (Fig. 1E). Subcellular localization analysis confirmed that SlMOC*^SP/ctp^-*eGFP was relocalized to the apoplastic space. In contrast, the full-length protein SlMOC1^FL^-eGFP localized predominantly to chloroplasts as predicted, the CTP-deleted variant SlMOC1*^ctp^*-eGFP displayed a diffuse localization pattern throughout the cytoplasm and nucleus (Fig. 1F).

### Extracellular localized SlMOC1 confers tomato resistance to bacterial wilt

**Figure 2:**
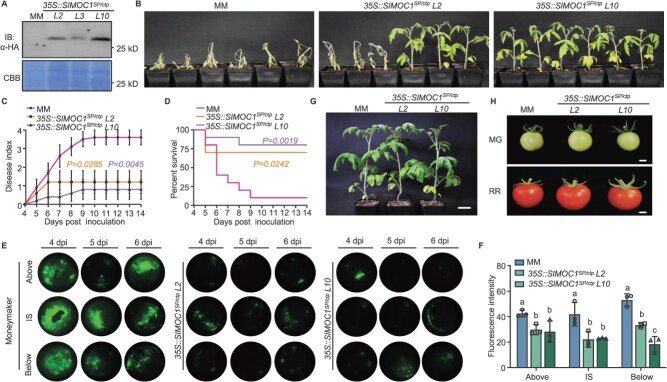
Constitutive expression of secretory MOC1 enhances tomato resistance to bacterial wilt. (A) Stable transgenic plants expressing SlMOC1*^ctp^* driven by the constitutive 35S promoter in the WT Moneymaker (MM) background were obtained. Total protein extracts from the *35S::SlMOC1^SP/ctp^* roots were analyzed by western blotting with an α-HA antibody. (B) Disease symptoms of the WT MM and *35S::SlMOC1^SP/ctp^*-overexpressing tomato upon GMI1000 infection by soil-drenching inoculation. Images were taken at 10 dpi. *L2* and *L10* are two lines of *35S::SlMOC1^SP/ctp^* transgenic plants. (C) Disease index of the inoculated plant symptom. Data are presented as mean values ± SEM (*n* = 10 individual plants). The *P*-value was calculated based on the AUDPC analysis. (D) The survival curve of the WT and *35S::SlMOC1^SP/ctp^* plants after infection with GMI1000. Statistical analysis of plant survival rate was performed using log-rank (Mantel–Cox) test. (E) The distribution and movement of GMI1000 in the xylem of MM and *35S::SlMOC1^SP/ctp^* lines. Stem sections collected from inoculation site (IS), above, and below were sampled after inoculation and imaged with a fluorescent dissecting microscope under white light and UV light. (F) Fluorescence intensity of GMI1000-GFP (*n* = 3 individual plants). Data are presented as mean values ± SD. Different letters indicate significant differences (one-way ANOVA, Tukey’s test, *P* < 0.05). (G) Growth morphology of *35S::SlMOC1^SP/ctp^* plants in budding stage. Ten-week-old tomatoes were photographed. Scale bars indicate 5 cm. (H) Fruits from MM and *35S::SlMOC1^SP/ctp^* plants at different developmental stages. Scale bars indicate 1 cm. MG, mature green; RR, red ripe. The above experiments were repeated three times with similar results.

To evaluate whether the secretory MOC1 can target the vascular biofilm and ultimately improve plant resistance. We generated a series of tomato lines expressing SlMOC1*^SP/ctp^*. Firstly, transgenic tomatoes expressing SlMOC1*^SP/ctp^*-HA under the control of constitutive 35S promoter were generated in the susceptible cultivar Moneymaker (Fig. 2A, Supplementary Fig. S1A). Following soil-drenching inoculation with *R. solanacearum* strain GMI1000, *35S::SlMOC1^SP/ctp^* lines exhibited milder wilting symptoms and lower disease index compared with the wild-type (WT) Moneymaker (Fig. 2B–D). To further explore whether the enhanced resistance of *35S::SlMOC1^SP/ctp^* lines is associated with the activation of plant immunity, we measured the expression levels of plant defense genes. The *35S::SlMOC1^SP/ctp^* plants did not exhibit induced expression of *SlPR1*, *SlERF2a*, and *SlEDR1* (Supplementary Fig. S1B–D), indicating that overexpression of SlMOC1^SP/ctp^ did not strengthen tomato immune responses. To visually monitor bacterial colonization within tomato vascular tissues, petioles were inoculated with a GFP-tagged GMI1000 and stem sections were imaged. Intense GFP fluorescence filled the xylem vessels of the WT plants, whereas *35S::SlMOC1^SP/ctp^* lines showed sharply reduced fluorescence both above and below the inoculation sites (Fig. 2E and F). Given the reported role of MOC1 in growth and chloroplast development in *Arabidopsis* [22], we assessed whether SlMOC1 engineering causes pleiotropic effects in tomato. Under greenhouse conditions, *35S::SlMOC1^SP/ctp^* plants flowered normally and did not exhibit significant differences in the height and fruit status of adult plants compared with WT (Fig. 2G and H). Together, these results indicate that expression of *35S::SlMOC1^SP/ctp^* enhances bacterial wilt resistance without detectable detrimental effects on plant growth.

**Figure 3:**
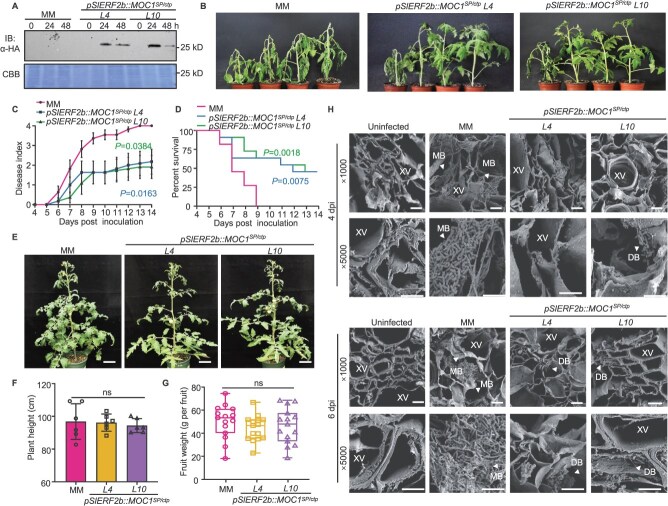
Inducible SlMOC1^SP/ctp^ expression confers tomato resistance to bacterial wilt. (A) Immunoblotting detection of MOC1^SP/ctp^ protein in *pSlERF2b:: MOC1^SP/ctp^*-overexpressing plants treated with GMI1000. *L4* and *L10* are two representative transgenic lines. Total proteins were analyzed with an α-HA antibody. The loading was shown by CBB staining. (B) Disease phenotype of the GMI1000-infected MM and *pSlERF2b::MOC1^SP/ctp^* plants. Photos were taken at 7 dpi. (C) Disease index of MM and *pSlERF2b::MOC1^SP/ctp^* plants post GMI1000 inoculation. Data points represent the average disease index ± SEM. The *P*-value was calculated based on the AUDPC analysis (*n* = 11 individual plants). (D) Survival ratio was analyzed by comparing the number of surviving plants to total plants from the data in (C). Statistical analysis of plant survival rate was performed using the log-rank (Mantel–Cox) test. (E) Growth phenotype of MM and the *pSlERF2b::MOC1^SP/ctp^* transgenic tomato plants in the greenhouse. Two-month-old plants were photographed. Scale bars indicate 10 cm. (F) Plant height of the transgenic tomato at breaker stage (*n* = 6 individual plants). Data are presented as mean values ± SD. (G) Mature fruit weight of *pSlERF2b::MOC1^SP/ctp^* plants (*n* = 15 individual fruits). (H) GMI1000 colonization in tomato vascular bundle sections after leaf cut-petiole inoculation. The representative SEM images showed the stem cross-sections of MM and *pSlERF2b::MOC1^SP/ctp^* plants at 4 and 6 dpi, respectively. Scale bars indicate 10 μm (×1000) and 5 μm (×5000). DB, developing biofilm; MB, mature biofilm; XV, xylem vessels. The significance of the difference in (F) and (G) was examined by one-way ANOVA analysis (*P* < 0.05). The above experiments were repeated three times with similar results.

To achieve the precise control spatiotemporally, we selected the promoter of *ethylene response factors 2b* (*SlERF2b*) gene, which is specifically activated upon *R. solanacearum* infection based on our previous studies, to drive the expression of *SlMOC1^SP/ctp^ in planta* [25]. The 2-kb promoter of *SlERF2b* was cloned from tomato genome and used to drive the expression of RsRuvC^SP^-HA in tomato hairy roots. Immunoblot analysis confirmed the pathogen-responsive activity of this promoter (Supplementary Fig. S2A). Hairy roots expressing *pSlERF2b::RsRuvC^SP^* displayed a lower disease index and higher survival rates compared to the empty vector controls (Supplementary Fig. S2B and C). We then generated *pSlERF2b::MOC1^SP/ctp^*-HA transgenic tomato plants and verified its expression induced by *R. solanacearum* (Fig. 3A; Supplementary Fig. S2D). To verify whether the expression of *pSlMOC1^SP/ctp^* driven by the inducible promoter can regulate the resistance to bacterial wilt, we inoculated plants with GMI1000 via the natural soil drenching. The *pSlERF2b::MOC1^SP/ctp^* lines exhibited delayed wilting symptoms relative to the WT (Fig. 3B). After 14 days post-inoculation (dpi), the disease incidence in *pSlERF2b::MOC1^SP/ctp^* lines was ~50% of that in the WT plants (Fig. 3C and D). The use of the inducible *SlERF2b* promoter did not impair normal plant growth and development (Fig. 3E–G).

To determine whether the reduced disease severity correlates with the inhibition of bacterial biofilm development in xylem vessels, we examined *R. solanacearum* biofilm in the stem tissues using scanning electron microscopy (SEM) following petiole inoculation. In WT plants, xylem vessels showed extensive biofilm formation and vascular occlusion by bacterial community with visible fibrous extracellular matrix indicative of mature biofilms. In contrast, *pSlERF2b::MOC1^SP/ctp^* plants exhibited only scattered adherent bacteria and minimal biofilm formation, resulting in markedly less vascular obstruction (Fig. 3H). Collectively, these data demonstrate that apoplastic delivery of MOC1 effectively disrupts biofilm formation in tomato xylem vessels, thereby enhancing resistance to bacterial wilt. Importantly, pathogen-inducible expression driven by the SlERF2b promoter achieves robust disease suppression without compromising plant growth, highlighting the superiority of this precision strategy for crop protection.

### Expression of secretory OsMOC1 confers resistance to bacterial blight in rice

**Figure 4:**
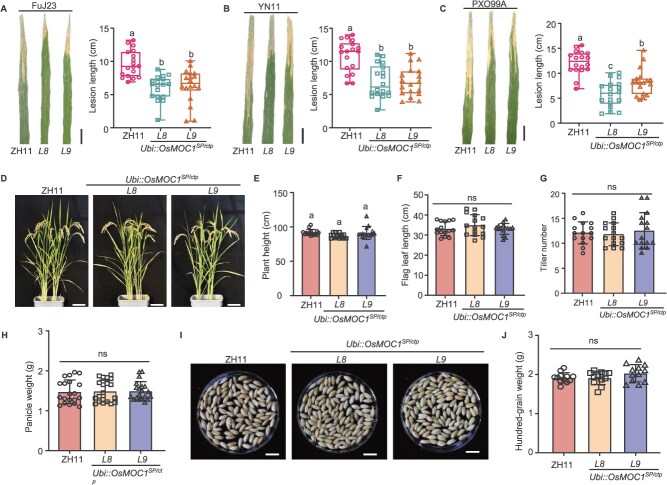
The engineered OsMOC1 promotes resistance of rice to bacterial blight. (A)–(C) Bacterial blight symptoms in ZH11 and *Ubi::OsMOC1^SP/ctp^*-overexpressing lines. *L8* and *L9* are two lines of *Ubi::OsMOC1^SP/ctp^* transgenic rice plants. Scale bars indicate 2 cm. The lesion length of *Ubi::OsMOC1^SP/ctp^* plants infected by *Xoo* strains from Yunnan (YN11) and Fujian (FuJ23) from China, and the *Xoo* standard strain PXO99A from Philippines was statistically analyzed (*n* = 18 individual leaves). Data are presented as mean ± SD. (D) Morphology of ZH11 and *Ubi::OsMOC1^SP/ctp^* plants at the heading stage. Scale bars indicate 10 cm. (E) Plant height of *Ubi::OsMOC1^SP/ctp^* lines after maturation in the field (*n* = 15 individual plants). Data are presented as mean values ± SD. (F) Flag leaf length of the WT and *Ubi::OsMOC1^SP/ctp^* rice at booting stage (*n* = 15 individual leaves). (G) Tiller number of WT and *Ubi::OsMOC1^SP/ctp^* plants (*n* = 15 individual plants). (H) Panicle weight of WT and *Ubi::OsMOC1 ^SP/ctp^* plants (*n* = 20 individual plants). (I) Images of seeds harvested from the experimental field. Scale bars indicate 10 mm. (J) Hundred-grain weight of seeds from WT and *Ubi::OsMOC1^SP/ctp^* (*n* = 12 individual plants). The significance of difference were examined by one-way ANOVA analysis (*P* < 0.05). The above experiments were repeated three times with similar results.

Bacterial blight caused by *X. oryzae* pv*.oryzae* (*Xoo*) is one of the most devastating bacterial diseases of rice worldwide [1, 26]. Biofilm formation is a critical virulence factor for *Xoo* to destroy rice vascular bundle [27, 28]. Evolutionary analyses indicate that MOC1 is conserved across crop species, and OsMOC1 is present in diverse rice varieties [18]. To investigate its potential functional role, we generated transgenic lines expressing *OsMOC1^ctp^* fused to the secretory peptide of OsPR1b under the constitutive maize *Ubiquitin 1* (*Ubi*) promoter in the ZH11 genetic background (Supplementary Fig. S3A). Following leaf-clipping inoculation under field conditions with three *Xoo* strains isolated from different regions in China and Philippines, these *Ubi::OsMOC1^SP/ctp^* lines exhibited significantly shorter lesion lengths than the WT ZH11 (Fig. 4A–C), indicating broad-spectrum resistance. Under field conditions, the agronomic traits of *Ubi::OsMOC1^SP/ctp^* progenies, including relative chlorophyll content (Supplementary Fig. S3B), plant height (Fig. 4D and E), flag leaf length (Fig. 4F), and tiller number (Fig. 4G) showed no significant differences with the WT plants. The transgenic lines also progressed normally throughout booting and grain-filling stages (Fig. 4H–J, S3C), demonstrating that apoplastic relocalization of OsMOC1 does not impose growth penalties.

### Effector-inducible OsMOC1 expression enhances resistance to bacterial blight

**Figure 5:**
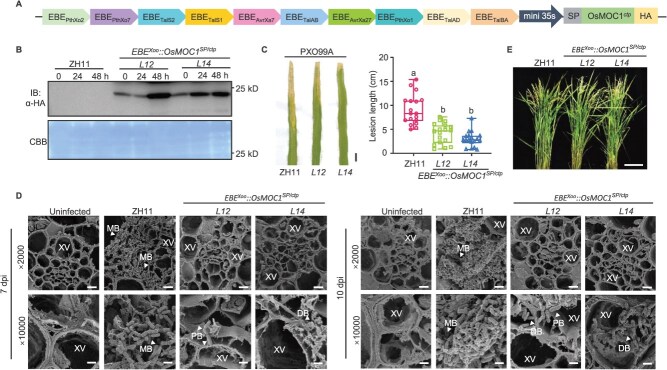
Driving OsMOC1 with EBE^Xoo^-stacking promoter boost resistance to bacterial blight in rice. (A) The schematic diagram of *EBE^Xoo^::OsMOC1^SP/ctp^* expression vector. The construction contains 10 EBE elements targeted by different TALEs. Secretory SP, OsMOC1*^SP/ctp^* coding sequence, and the HA-tag were shown with different colors. (B) Immunoblotting analysis of OsMOC1^SP/ctp^ protein in leaves of *EBE^Xoo^::OsMOC1^SP/ctp^* plants treated with PXO99A. *L12* and *L14* are two lines of *EBE^Xoo^::OsMOC1 ^SP/ctp^* transgenic rice plants. Total proteins were analyzed with an α-HA antibody. The loading was shown by CBB staining. (C) Bacterial blight symptoms and lesion lengths of ZH11 and *EBE^Xoo^::OsMOC1^SP/ctp^* leaves inoculated with *Xoo* PXO99A strain (*n* = 15 individual leaves). The WT ZH11 was used as the control group. Scale bars indicate 2 cm. Different letters indicate significant differences (one-way ANOVA, Tukey’s test, *P* < 0.05). (D) The vascular bundles in leaves of *EBE^Xoo^::OsMOC1^SP/ctp^* transgenic plants after PXO99A inoculation by SEM observation. The representative images show the leaf sections of the WT ZH11 and *EBE^Xoo^::OsMOC1^SP/ctp^* plants at 7 and 10 dpi. Scale bars indicate 5 μm (×2000) and 1 μm (×10 000). DB, developing biofilm; MB, mature biofilm; PB, planktonic bacteria; XV, xylem vessels. (E) Gross morphology of the WT and *EBE^Xoo^::OsMOC1^SP/ctp^* plants at heading stage. Scale bars indicate 10 cm.

*Xanthomonas.*spp pathogens deploy transcription activator-like effectors (TALEs) to induce the expression of susceptibility genes by binding to effector-binding elements (EBEs) in the promoter regions, which facilitate infection [29, 30]. Previous studies have found that EBE-stacking strategy can improve the resistance of rice to bacterial blight and bacterial leaf streak [12, 31]. To exploit this pathogen-specific response mechanism, we assembled a synthetic EBE array corresponding to 10 conserved TALEs in *Xoo* strains from Asia and placed it upstream of a minimal 35S promoter [32–35], finally generating the *Xoo*-activated EBE-tandem promoter (Fig. 5A; Supplementary Table S2). Immunoblot analysis confirmed the rapid activation of EBE-tandem promoter upon *Xoo* strain PXO99A infection (Fig. 5B).

Transgenic rice expressing *EBE^Xoo^::OsMOC1^SP/ctp^* was generated and the resistance was evaluated at the tillering stage by leaf-clipping inoculation with PXO99A under field conditions. The lesion length of *EBE^Xoo^::OsMOC1^SP/ctp^* was significantly reduced by >65% compared WT ZH11 (Fig. 5C). To visualize *Xoo* colonization, we examined leaf sections at the junction of diseased and healthy tissue from WT ZH11 and *EBE^Xoo^::OsMOC1^SP/ctp^* at 7 and 10 dpi by SEM. At both time points, xylem vessels of WT leaves were densely filled with bacteria embedded in the thick biofilm matrix. In contrast, vessels of *EBE^Xoo^::OsMOC1^SP/ctp^* contained only a small number of bacterial aggregates and a markedly thinner biofilm (Fig. 5D), indicating impaired multiplication and vascular colonization. Field observations revealed no significant difference in the growth between *EBE^Xoo^::OsMOC1^SP/ctp^* plants and WT ZH11 (Fig. 5E). Relative chlorophyll content remained unchanged under natural field conditions (Supplementary Fig. S4A). Key agronomic traits, such as plant height (Supplementary Fig. S4B), panicle development (Supplementary Fig. S4C and D), and seed weight (Supplementary Fig. S4E and F) of *EBE^Xoo^::OsMOC1^SP/ctp^* lines were comparable to those of the WT plants.

## Discussion

**Figure 6:**
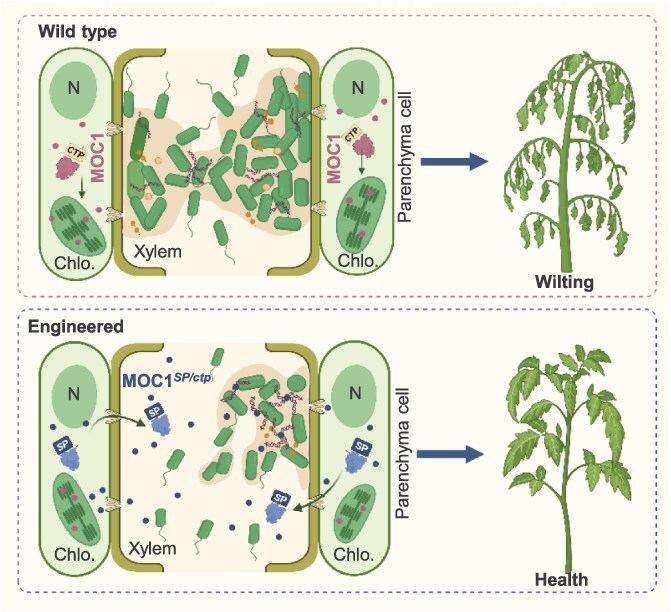
Working model for relocalization of plant MOC1 against vascular bacterial pathogen. In the WT background, MOC1 is normally transported into chloroplast, where it functions in DNA recombination and doesn’t target bacterial biofilm in the xylem. Under the engineered condition, the CTP is replaced with an SP, which results in MOC1 being secreted into the extracellular space. Once in the xylem, MOC1 directly disrupts the eDNA lattice of the biofilm, suppresses pathogen colonization, and thereby enhance plant vascular resistance. MOC1 represents the full-length native protein, MOC1*^SP/ctp^* represents the engineered secreted variant. N indicates the nucleus and Chlo. indicates chloroplasts.

Plant vascular tissue has long been considered as a vulnerable site due to the lack of effective immunity, making it susceptible to pathogen colonization [36]. Bacterial pathogens such as *R. solanacearum* and *Xoo* exploit robust biofilms to persist within xylem vessels and cause devastating diseases [3, 28, 37]. In the WT plants, MOC1 proteins are transported into the chloroplast and could not contact bacterial biofilm in the xylem (Fig. 6). To enhance vascular defense by targeting these biofilms, we designed a strategy to relocalize the plant HJ resolvase MOC1 to the extracellular space, thereby disrupting pathogenic biofilms inside the xylem (Fig. 6). We validated this approach in both dicot (tomato) and monocot (rice) crops, demonstrating that engineered plants exhibit broad-spectrum resistance. Furthermore, we employed two distinct expression strategies to optimize MOC1 expression. A constitutive promoter was first tested to confirm the function of SlMOC1*^SP/ctp^*. Subsequently, pathogen-inducible promoters were applied to achieve precisely spatial and temporal control, ensuring MOC1 protein expression only upon pathogen invasion [25, 38]. Transgenic lines showed effective restriction of bacterial colonization and disease progression, without any observable penalty on plant growth and development. These results demonstrate that engineering a conserved host protein for apoplastic secretion provides a powerful and versatile approach to combat biofilm-dependent vascular pathogens.

Although the high conservation of MOC1 among different plant species and the central role of biofilms in bacterial pathogenesis make this strategy widely applicable [14, 18, 23], the potential impacts on the microbial community should be taken into account during the crop improvement process. Based on this, we utilized promoters that locally elevate MOC1 concentration in the apoplast upon pathogen invasion or specific induction, thereby inhibiting pathogens. In the noninfected state, the basal expression level of MOC1 is likely insufficient to exert significant pressure on the microbial community. Additionally, we did not observe a significant growth disadvantage in transgenic plants during soil culture, which indirectly indicates that their rhizosphere microecology remains unimpaired. In the future, it is necessary to further evaluate the tolerance of the modified germplasm to the environment and monitor its effects on nontarget organisms such as soil microorganisms and endophytic bacteria during an extended time course.

Our study provides an efficient platform for further exploration of disease resistance gene resources for plant immunity enhancement. The expression of secreted MOC1 protein conferred resistance to both *Ralstonia* and *Xoo*, indicating its potential as a general tool for targeting biofilm-dependent bacterial pathogens. In the future, it would be worthwhile to use CRISPR-based technology to replace the chloroplast transit peptide with a secretion signal peptide *in situ*, thereby generating DNA-free edited plants with improved vascular immunity.

## Materials and methods

### Plant and bacterial materials

Tomato (*Solanum lycopersicum*) variety Moneymaker was used in this study as the WT. Tomato was grown in soil substrates (Pindsrup) at 22°C, 45% relative humidity, and 75 μE/m2/s1 (T5 LED tube lights, 4000 K) with 12-h light/12-h dark cycles. The rice variety Zhonghua11 (ZH11) was used for transformation in most of this study. Rice plants grew in the natural environment of the experimental field and the growth chamber for inoculation with *X. oryzae* pv*. oryzae* (*Xoo*).

*R. solanacearum* strain GMI1000 was grown on Casamino Acids-Peptone-Glucose (CPG) solid or liquid medium at 28°C. *Xoo* strains were grown at 28°C in Nutrient Broth (NB) medium. *Agrobacterium tumefaciens* and *A. rhizogenes* strains carrying different plasmids were grown on Luria–Bertani (LB) medium with corresponding antibiotics at 28°C.

### Pathogen infection assays

For soil-drench inoculation with *R. solanacearum*, the root systems of the tested plants were treated with 10 ml of bacterial suspension adjusted to OD_600_ 0.1. After inoculation, the plants were kept in a growth chamber under controlled conditions: 28°C, 75% humidity, and a 12-h light/12-h dark cycle for ~14 days. Disease severity was evaluated on a 0–4 scale, where 0 indicated no symptoms; 1 denoted 1%–25% leaf wilting; 2 represented 26%–49%; 3 corresponded to 50%–74%; and 4 indicated complete wilting. Bacterial virulence was quantified by calculating the area under the disease progression curve (AUDPC), which was analyzed statistically via linear mixed-effects models (LMMs) in R, as previously outlined [39]. For survival analysis, disease index was converted into binary values: scores <2 were classified as ‘0’, while scores of ≥2 were classified as ‘1’ at each observation time. Statistical analysis was performed using a Log-rank (Mantel–Cox) test of the Kaplan–Meier estimate.

For cut-petiole inoculation with *R. solanacearum-*expressing GFP, the petiole of the first round of leaves was cut and inoculated by applying a 2-μl droplet of bacterial suspension (1 × 10^6^ CFU/ml) to the wound, as previously described [37]. Inoculated plants were maintained in a growth chamber after the bacterial droplet was fully absorbed.

Rice flag leaves were inoculated with *Xoo* strains at the booting (panicle development) stage by the leaf-clipping method. *Xoo* strains used in this study included the Philippine strain PXO99A and the Chinese strains YN11 and FuJ23. The bacterial suspension was diluted to 5 × 10^6^ cfu/ml. Disease was scored by measuring the lesion length at 14 dpi.

### Observation and quantitative analysis of eDNA in biofilm

To measure the eDNA amount, *R. solanacearum* GMI1000 was diluted to OD _600_ = 0.1, mixed with different concentrations of SlMOC1 and cocultured at 28°C for 48 h. The eDNA content was detected using the dsDNA HS Assay Kit (Yeasen, 12640ES76). All test samples were mixed with the working solution and stained in the dark for 5 min. Fluorescence intensity was measured using the multimode reader platform (Tecan, Spark 10 M). The standard curve was generated using a dsDNA standard with a known concentration to calculate the content of eDNA.

To observe the eDNA network structure within biofilms, PicoGreen staining was conducted as follows. Cover glasses were placed at the bottom of cell culture plates, and a bacterial suspension adjusted to OD_600_ = 0.2 was added to each well. Biofilms were allowed to form on the cover glasses at 28°C for 48 h. The cell culture plates were rinsed with 1× PBS and eDNA was stained with PicoGreen (Yeasen, 12641ES02) solution in the dark for 15 min. Fluorescent signals were detected using a laser scanning confocal microscope (Leica, SP8), and fluorescence intensity was quantified with ImageJ.

### Transient expression and protein subcellular localization in *N. benthamiana*

The binary vectors were transformed into *A. tumefaciens* and *A. rhizogenes* by electroporation. Before infiltration, the transformed Agrobacteria were induced with 200 μM acetosyringone (Sigma, Cat. D134406) for 3 h. The bacterial suspension was adjusted to an OD_600_ of 1.0 for infiltration. Subsequently, samples were collected 48 h postinfiltration (hpi) for further analysis based on experimental needs.

The subcellular localization of different SlMOC1 versions in tobacco leaves was determined by *Agrobacterium*-mediated transient transformation. GFP fluorescence signals in the *N. benthamiana* leaf discs were examined at 48 hpi using confocal microscopy (Leica, SP8). Green fluorophores were excited at 488 nm and detected through 510 nm filter.

### RNA isolation and RT-qPCR analysis

Total RNA was isolated from tomato with Trizol reagent (TIANGEN, Cat. DP424). Total RNA was reverse-transcribed to synthesize first-strand cDNA at 50°C for 30 min after treatment with RNase-free DNase I at 42°C for 5 min (Vazyme, Cat. R223-01). Reverse transcription quantitative PCR (RT-qPCR) analysis was carried out using SYBR Green Supermix (Vazyme, Cat. Q711-02) with gene-specific primers (Supplementary Table S3). RT-qPCR data were collected using the Analytik Jena qPCRSoft 4.1 software. The expression of each tomato gene was normalized to that of *SlACTIN2*.

### Observation of bacterial biofilm in xylem by SEM

For the preparation of tomato samples, after petiole inoculation of 4-week-old WT and transgenic tomato with *R. solanacearum* and using uninoculated plants as a control, stem segments were harvested at the onset of symptoms (Disease Index = 1). For the preparation of rice samples, the rice leaves at the booting stage were inoculated with *Xoo* by the leaf-clipping method. The leaf samples at the junction of disease and health were collected 7 and 10 dpi, respectively. At this time, it can be seen that the lesion began to expand.

All samples were surface-sterilized and fixed in 2.5% glutaraldehyde (Solarbio, Cat#P1126). The samples were then rinsed three times with 0.1 M phosphate buffer (pH 7.0) and postfixed with 1% osmic acid. Subsequently, they were dehydrated through a graded ethanol series (30%, 50%, 70%, 80%, 90%, 95%, and 100%), followed by treatment with a 1:1 (v/v) mixture of ethanol and isoamyl acetate. Finally, the pure isoamyl acetate was dried at the critical point. The samples were observed under scan electronic microscopy (Hitachi, Reguius 8100).

### Agrobacterium-mediated plant transformation

For tomato genetic transformation, the expression vector *pCAMBIA2300-35S::SlMOC1^SP/ctp^-HA* or *pCAMBIA2300-pSlERF2b::MOC1^SP/ctp^-HA* was first introduced into *A. tumefaciens* strain GV3101. Putative transformants were selected from kanamycin-containing medium. Those regenerants that successfully developed roots were further analyzed by immunoblotting with an α-HA antibody (Roche, Cat. 12 013 819 001) to confirm target protein expression.

For transient expression in tomato roots, the binary construct *pCAMBIA2300-pSlERF2b::RsRuvC^SP^-HA* was introduced into *A. rhizogenes* strain MSU440. Tomato variety Moneymaker was then transformed following established methods [40]. Briefly, 2-week-old tomato seedlings were prepared by excising the bottom of the hypocotyl. Bacteria harvested from the plate were applied to the wound sites. The inoculated seedlings were transferred to sugar-free one-half MS medium and covered with a semicircular filter paper. After 7 days, the emerging nontransformed hairy roots were excised with a sterile scalpel. The plants were then transferred to one-half MS medium containing 25 μg/ml kanamycin for selection. Finally, the rooting plants screened on the plate were induced by *R. solanacearum* GMI1000 strain. After induction, tomato roots were collected for immunoblot detection.

For *Oryza sativa* transformation, OsMOC1*^SP/ctp^* driven under Ubiquitin1 (Ubi) or EBE promoter was fused with HA and inserted into *pRHVcHA* vector to construct OsMOC1*^SP/ctp^*-HA overexpression vector [41]. These constructs were transferred into *A. tumefaciens* strain EHA105, which were further transformed into rice calli from mature embryos of ZH11. Immunoblotting was used to screen plants with OsMOC1*^SP/ctp^* protein expression for propagation.

### Statistical analysis

Statistical analyses were conducted using GraphPad Prism 8, with quantification data presented as mean ± standard deviation (SD) or standard error of the mean (SEM). Differences between groups were assessed by a two-tailed Student’s *t*-test or by one-way analysis of variance (ANOVA), followed by Tukey’s *post hoc* test. Statistical significance was set at *P* < 0.05, and ns indicated no significance. The figure legends specify the number of biological replicates and independent experiments. All experiments were independently repeated at least three times with consistent results.

### Accession numbers

Sequence data in this article can be found in the *R. solanacearum* Database, NCBI website, and Sol Genomics Network website under the following accession numbers: SlMOC1 (Solyc01g008700), SlPR1 (Solyc01g106620), OsPR1b (Os01g28450), SlERF2b (Solyc04g051360), SlACTIN2 (Solyc11g005330), OsMOC1 (Os01g16340), RuvC (RSc0503)**.** All data supporting the findings of this study are available within the Article and its Supplementary Information or from the corresponding author upon reasonable request.

## Supplementary Material

Web_Material_uhag046

## Data Availability

All the data supporting the findings of this study are included in this article or as supporting information.
