# Supplementary material for: Chloroplast-to-apoplast relocalization of MOC1 strengthens plant vascular immunity
Source: Hortic Res. 2026 Feb 19;13(5):uhag046. doi: 10.1093/hr/uhag046 (PMC13222483; doi:10.1093/hr/uhag046)
Supplement: Web_Material_uhag046 [file web_material_uhag046.zip › MOC1 Supplementary figures 0127.docx]

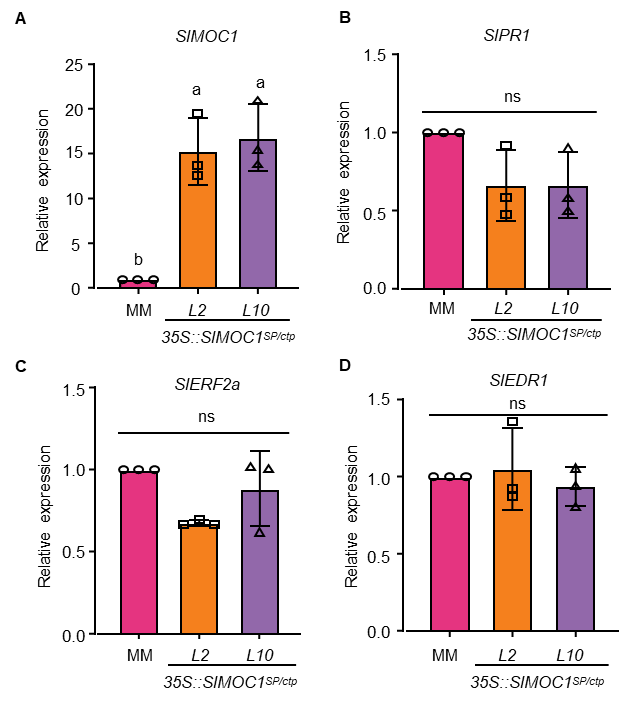


**Supplementary Figure S1.** The expression level of SlMOC1 and defense -related genes in transgenic tomato plants. (A) Expression levels of the SlMOC1 were measured in the stem tissues of *35S::SlMOC1^SP/ctp^*. (B) The expression of defense genes was not induced in *35S::SlMOC1^SP/ctp^* plant. Total RNA was isolated from tomato stems, and the expression levels of genes were quantified via reverse transcription quantitative real-time PCR (RT-qPCR), with *SlACTIN2* serving as the reference gene for normalization. Data are presented as mean values ± SD (n=3 technical repeats). The wild-type Moneymaker (MM) were designated as the control group. The significance of the difference were examined by one-way ANOVA analysis (*P* < 0.05). The above experiments were repeated three times with similar results.


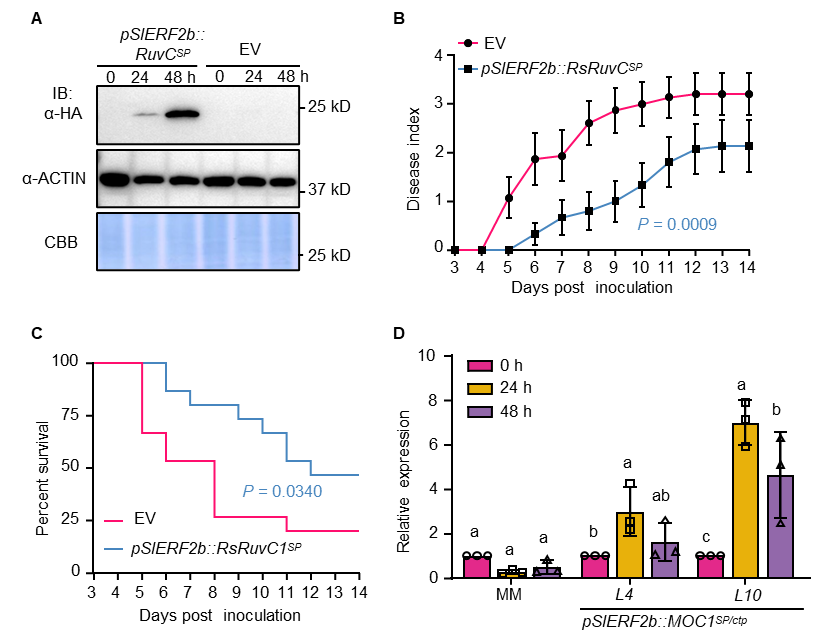
**Supplementary Figure S2.** Verification of expression pattern and function of the *R. solanacearum-*inducible *SlERF2b* promoter. (A) The expression of *RuvC^SP^* protein is induced by GMI1000 infection in the hairy roots of *pSlERF2b::RuvC^SP^* transformed tomato plants. Total proteins were analyzed by immunoblotting with an α-HA antibody. The protein level of tomato ACTIN was used as an internal control. Plants transformed with an empty binary vector (EV) were used as controls. (B)-(C) Disease index (B) and survival rate (C) of *p**SlERF2b::RuvC^SP^* and the control plants after inoculation with GMI1000 (n=15 individual plants). Data are presented as mean values ± SEM in (B). Statistical analysis of plant survival rate in (C) was performed using a Log-rank (Mantel-Cox) test. (D) Verification of GMI1000-induced expression of *SlMOC1* in Moneymaker (MM) and *pSlERF2b::RuvC^SP^* transgenic tomato lines. Total RNA was extracted from tomato roots. Expression levels of *SlMOC1* in RT-qPCR analysis were normalized to *SlACTIN2*. Data are presented as mean values ± SD (n=3 technical repeats from one independent experiment). The significance of the difference in (D) was examined by one-way ANOVA analysis (*P* < 0.05). The above experiments were repeated three times with similar results.


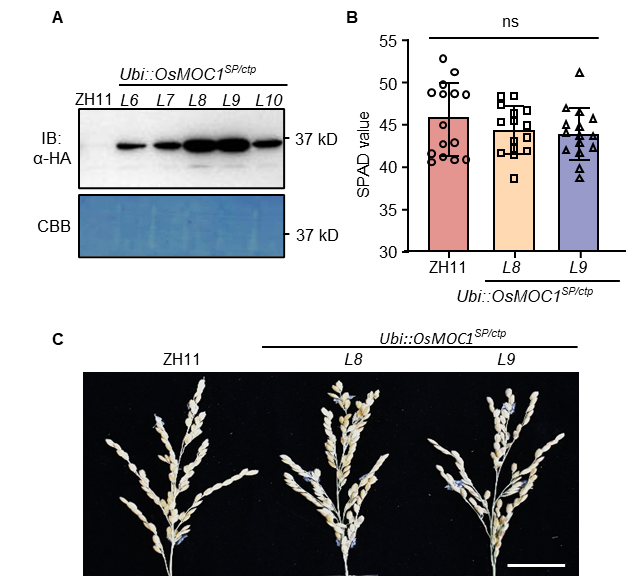


**Supplementary Figure S3.** Phenotypical characterization of *Ubi::OsMOC1^SP/ctp^* transgenic rice. (A) Detection of OsMOC1*^SP/ctp^* protein expression in *Ubi::OsMOC1^SP/ctp^* transgenic rice lines. Total protein was extracted from leaves and analyzed by immunoblotting using an α-HA antibody. The loading was shown by Coomassie brilliant blue (CBB) staining. (B) Relative chlorophyll content in wild-type ZH11 and *Ubi::OsMOC1^SP/ctp^* plants. Data are presented as mean values ± SD (n=15 individual leaves). The significance of the difference was examined by one-way ANOVA analysis (*P* < 0.05). (C) Panicle morphology of the WT and *Ubi::OsMOC1^SP/ctp^*. Scale bars indicate 3 cm. The above experiments were repeated three times with similar results.

**
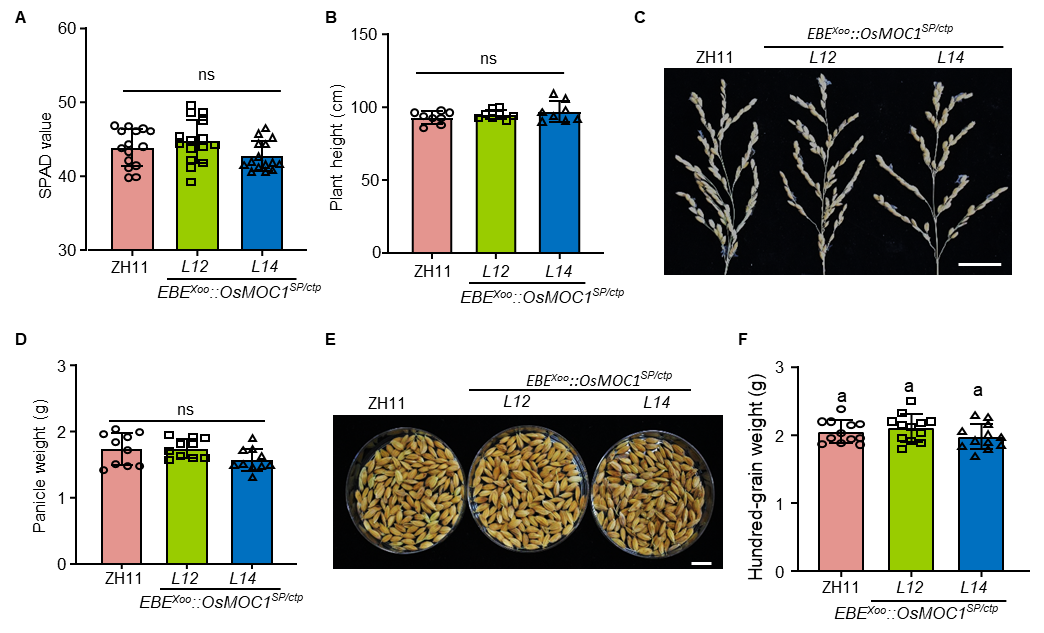
**

**Supplementary Figure S4.** The developmental phenotypes of *EBE^Xoo^-MOC1^SP/ctp^* transgenic rice. (A) Relative chlorophyll content in WT and *EBE^Xoo^::OsMOC1^SP/ctp^* plants (n=15 individual leaves). (B) Plant height of mature WT and *EBE^Xoo^::OsMOC1^SP/ctp^* plants grown under field conditions (n=8 individual plants). (C) Panicle morphology of the WT and *EBE^Xoo^::OsMOC1^SP-△ctp^* plants. Scale bars indicate 3 cm. (D) Statistical analysis of panicle weight in WT and *EBE^Xoo^::OsMOC1^SP/ctp^* plants (n=10 individual plants). (E) Grain phenotype of WT and *EBE^Xoo^::OsMOC1^SP/ctp^* plants harvested from the experimental field. Scale bars indicate 10 mm. (F) Hundred-grain weight of seeds. Data are presented as mean values ± SD (n=12 individual plants). Data in (A), (B), (D), and (F) are presented as mean values ± SD. The significance of the difference was examined by one-way ANOVA analysis (*P* < 0.05). The above experiments were repeated three times with similar results.
