# Supplementary material for: Chloroplast-to-apoplast relocalization of MOC1 strengthens plant vascular immunity
Source: Hortic Res. 2026 Feb 19;13(5):uhag046. doi: 10.1093/hr/uhag046 (PMC13222483; doi:10.1093/hr/uhag046)
Supplement: Web_Material_uhag046 [file web_material_uhag046.zip › MOC1 Supplementary Tables 1-3 0119.docx]

**Supplementary** **Table S1. The DNA sequences of the secretory signal peptides used in this study**

| Names | DNA Sequences |
| --- | --- |
| SlPR1^SP^ | ATGGGATACTCCAATATTGCTTTAATCATTTGTTTCCTTACCTTTGCTATATTTCACTCATCACAAGCTCAAAAC |
| OsPR1b^SP^ | ATGGAGGTATCCAAGCTGGCCATTGCTTTGGCCATGGTAGCCGCCATGGCACTCCCCTCCCAAGCT |

**Supplementary** **Table S2 The DNA sequences of the effector binding elements (EBEs) for promoter design in this study**

| Names | DNA Sequences |
| --- | --- |
| EBE_PthXo2_ | ATAAAAGCACCACAACTCCCTT |
| EBE_PthXo7_ | ATAATCCCCAAATCCCCTCCTC |
| EBE_TalS2_ | ATATTCATCGTTTCTCCAGT |
| EBE_TalS1_ | ATAGGGGGCAATCACAAACCCAC |
| EBE_AvrXa7_ | ATAAACCCCCTCCAACCAGGTGCTAA |
| EBE_TalAB_ | ATAAATCGGCGCGGCGCT |
| EBE_AvrXa27_ | AGCTAGGGGAATCCATG |
| EBE_PthXo1_ | GCATCTCCCCCTACTGTACACCAC |
| EBE_TalAD_ | CCATAGTACGCGCGCGAGGGGAAG |
| EBE_TalBA_ | AGCTAGCTTAGCCCCT |

**Supplementary** **Table S3 Primers used in this study**

| Primer names | DNA Sequences (5’-3’) |
| --- | --- |
| SlMOC1-SpeI-FLFP | CGGACTAGTATGGAATCAATTATCTTACAAACTC |
| SlMOC1-StuI-FLRP | AAAAGGCCTGTTGTTAAGCGCTGGAGTTG |
| SlMOC1ctp-SpeI-FP | CGGACTAGTGCCATTACCACAGATTCCC |
| SlERF2b-promoter-SpeI-FP | CCCAAGCTTGAATTATCTTATTCGTGGTACT |
| SlERF2b-promoter-BamHI-RP | ATTGGAGTATCCCATGGATCCGTATATTTGTATATATGAAATGTTTATTTT |
| OsMOC1*^ctp^*-StuI-FP | AAAAGGCCT GCCGCCGAGACGAGGCCCAC |
| OsMOC1-KpnI-FLRP | CGGGGTACC TAATGTTTTGCTGAACTCCCCTG |
| RsRuvC-FLFP-SpeI | CGGACTAGTATGCGCATCCTCGGCATCG |
| RsRuvC-FLRP-StuI | AAAAGGCCTGCCGACCAGCCGGCCG |
| SlMOC1-qRT-FP | CCAAATCAAACTCCTCAGGTGT |
| SlMOC1-qRT-RP | CCACCATCCCTGCTTACCA |
| SlPR1_qPCR_FP | ACTCAAGAGCTGGTAATTGCAAC |
| SlPR1_qPCR_RP | TTTCGATACCCACAATTGCACGG |
| SlERF2a_qPCR_FP | TATGCACAATTACTTCGCGATG |
| SlERF2a_qPCR_RP | TTCACTAGGTGGTCCAGTACTA |
| SlEDR1_qPCR_FP | TTAAGATCGTTAACCGGATCGT |
| SlEDR1_qPCR_RP | CCGATATCCACGTCGATTTCTA |
| SlACTIN2_qPCR_FP | GTATGTTGCTATTCAGGCTGTG |
| SlACTIN2_qPCR_RP | GCAAAGCATAACCCTCGTAAAT |
