# Supplementary figures and images for: Chloroplast-to-apoplast relocalization of MOC1 strengthens plant vascular immunity
Source: Hortic Res. 2026 Feb 19;13(5):uhag046. doi: 10.1093/hr/uhag046 (PMC13222483; doi:10.1093/hr/uhag046)

**Fig S1**

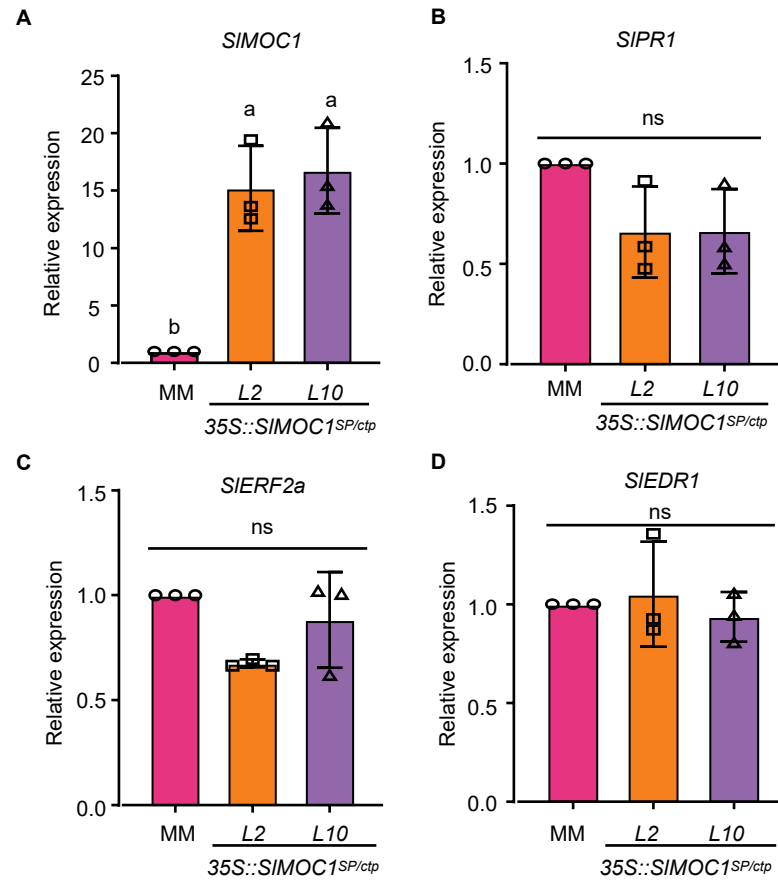

Supplement: Web_Material_uhag046 [file web_material_uhag046.zip › Sup Figure 1.pdf]

**Fig S2**

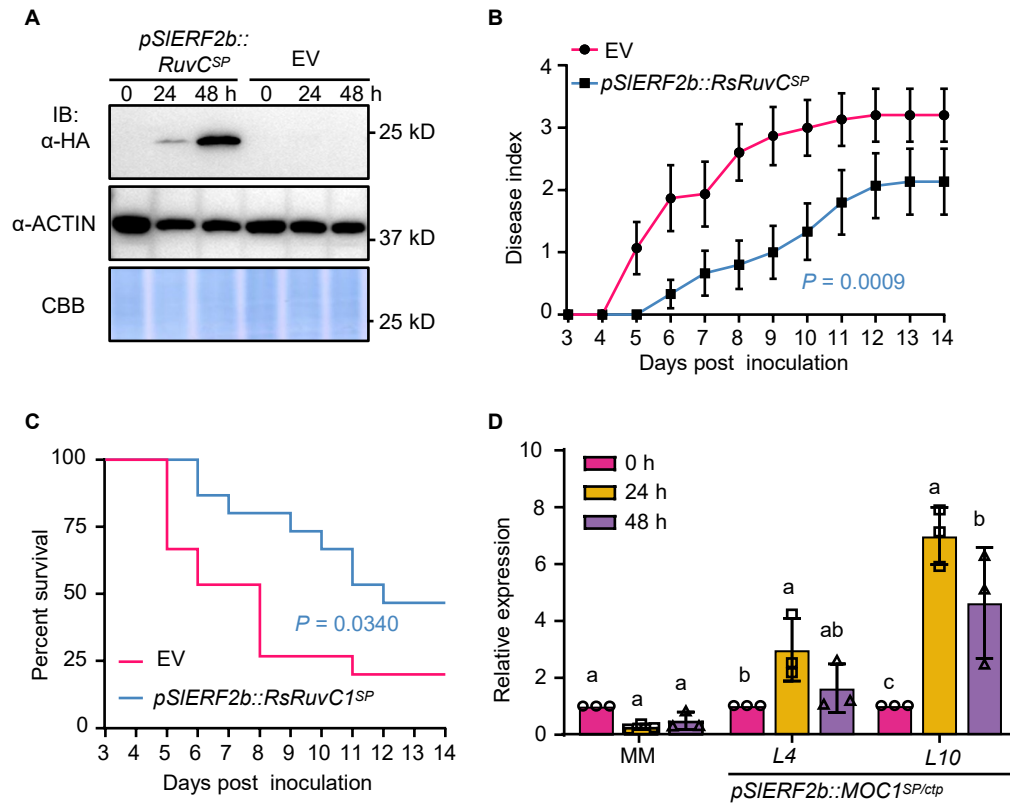

Supplement: Web_Material_uhag046 [file web_material_uhag046.zip › Sup Figure 2.pdf]

**Fig S3**

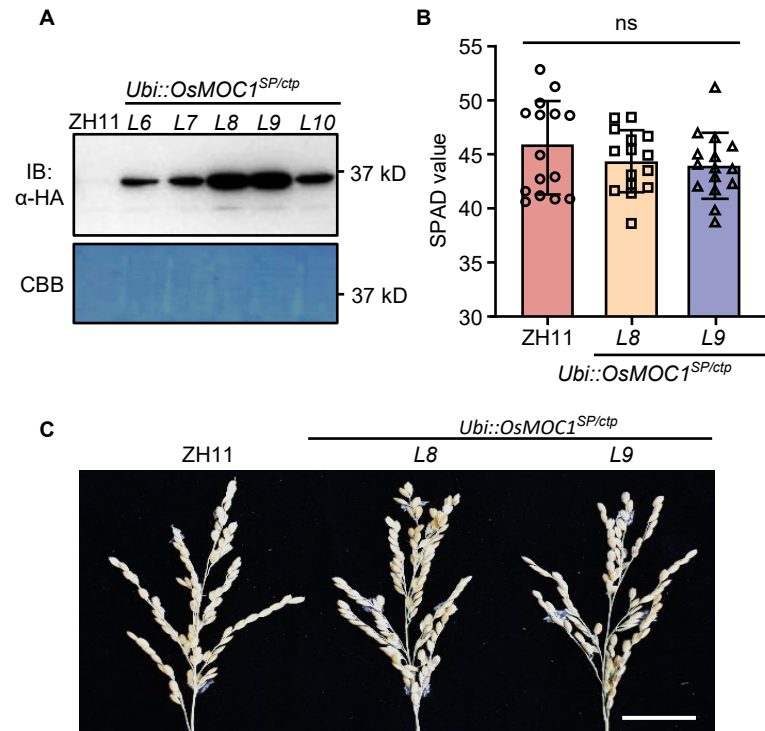

Supplement: Web_Material_uhag046 [file web_material_uhag046.zip › Sup Figure 3.pdf]

**Fig S4**

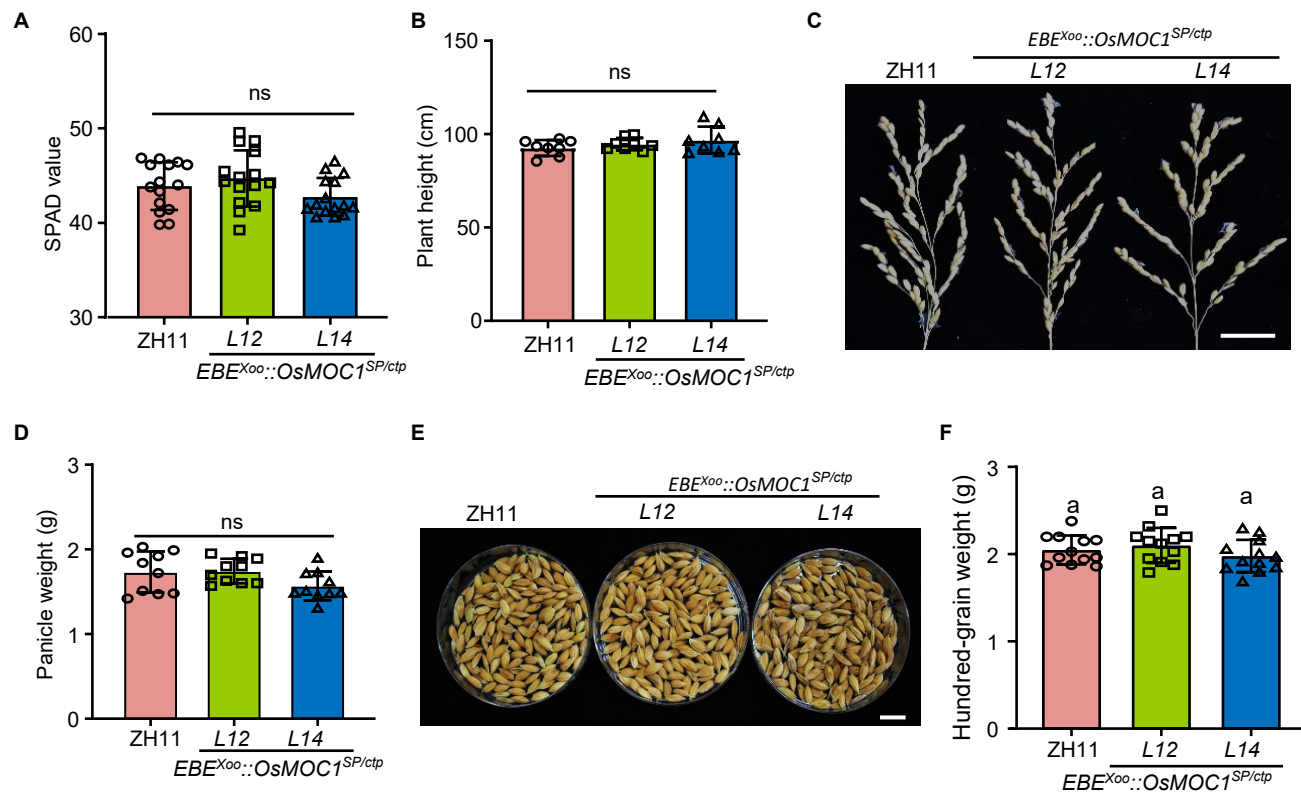

Supplement: Web_Material_uhag046 [file web_material_uhag046.zip › Sup Figure 4.pdf]
